# Diversity of gammacoronaviruses and deltacoronaviruses in wild birds and poultry in Russia

**DOI:** 10.1038/s41598-022-23925-z

**Published:** 2022-11-12

**Authors:** Vasily Marchenko, Alexey Danilenko, Natalia Kolosova, Maria Bragina, Marina Molchanova, Yuliya Bulanovich, Vladimir Gorodov, Sergey Leonov, Andrey Gudymo, Galina Onkhonova, Svetlana Svyatchenko, Alexander Ryzhikov

**Affiliations:** 1 State Research Center of Virology and Biotechnology Vector Rospotrebnadzor, Koltsovo, 630559 Russia; 2 Siberian Federal Scientific Centre of Agro-BioTechnologies, RAS, Novosibirsk, Russia

**Keywords:** Virology, Viral reservoirs, Next-generation sequencing

## Abstract

Coronaviruses of the genera *Gammacoronavirus* and *Deltacoronavirus* are globally widespread and circulate primarily in wild and domestic birds. Prior studies have established frequently occurring crossover events from avian to mammalian reservoirs. However, there is limited understanding of the diversity and geographical distribution of coronaviruses among birds. In this study, the surveillance of coronaviruses in birds in Russia during 2020 revealed the presence of coronaviruses in 12% of samples from birds. Targeted NGS approach was used for the evaluation of genetic diversity based on RdRp gene. While gammacoronviruses were found in both wild birds and poultry, deltacoronaviruses were found in wild birds only and represent the first detections for Russia. A number of cases with the simultaneous detection of gamma- and deltacoronaviruses in one bird was reported. The results of this study highlight the importance of further research concerning the spread and diversity of coronaviruses among birds within and migrating throughout the territory of Russia across the globe.

## Introduction

Coronaviruses (CoVs) are an important subject of research considering they often serve as infectious agents that are capable of causing disease in human and animal hosts. Susceptible taxa include a wide range of mammals, as well as various species of wild and domestic birds. Currently there is a very limited understanding of the diversity of coronaviruses, their transmission mechanisms and the role of individual species in disparate populations. Coronaviruses are common name of representatives of family *Coronaviridae,* suborder *Cornidovirineae*, order *Nidovirales.* According to the International Committee on Taxonomy of Viruses (ICTV), the family *Coronaviridae* is classified into three subfamilies, one of which is subfamily *Orthocoronavirinae* and includes four genera of viruses. The *Alphacoronavirus, Betacoronavirus, Gammacoronavirus and Deltacoronavirus* are of particular importance for agriculture and public health. Of the four genera of CoVs, the representatives of genera *Alphacoronavirus* and *Betacoronavirus* are considered to circulate most commonly among mammals, while birds serve as main hosts of viruses of genera *Gammacoronavirus* and *Deltacoronavirus*^1–4^.

The genus *Gammacoronavirus* is classified into three subgenera, two of which, subgenus *Brangacovirus* and subgenus *Igacovirus*, were identified only in birds. The subgenus *Cegacovirus* is represented by *Beluga whale coronavirus SW1,* which was isolated from a marine mammal^5^. The well-known and widespread representatives of the genus *Gammacoronavirus* are the *Avian coronavirus* (AvCov) and *Avian coronavirus 9203* (AvCov9203), subgenus *Igacovirus.* These two species represent a group of viruses that includes all infectious bronchitus virus (IBV) genotypes as well as other genetically similar viruses. IBV is the causative agent of avian infectious bronchitis in chickens, while other viruses of this group cause disease in turkeys, guinea fowls and other species. These viruses are ubiquitous in agricultural settings, and are especially widespread in regions where commercial poultry farming. AvCovs cause significant economic damage due to the infection associated with serious respiratory, genitourinary and digestive problems in poultry^6,7^. In addition, there are many reports of the isolation of gammacoronaviruses (gammaCoVs) from other bird species, indicating the ability of the virus to infect a wider range of hosts. Thus, studies conducted in Brazil and China have shown the isolation of IBV from wild peacocks and Rock pigeons (*Columba livia*)^8,9^. In another European study, similar viruses have been isolated from the droppings of healthy wild ducks and Whooper swans (*Cygnus cygnus*). These findings suggest interspecies transmission of the IBV viruses among domestic and wild birds, and therefore possible spread over considerable distances by migrating birds^10^. A recent update of the ICTV (2019) includes *Duck Coronavirus 2714* (DCoV) ratified species in the subgenus *Igacovirus*, which represent a large group of gammaCoVs commonly found in wild birds. There are reports that viruses from this group are able to cross interspecies barriers and infect poultry species commonly including domestic ducks, and chickens in some rare cases. Thus, there is a considerable diversity of gammaCoVs in domestic and wild birds that presents a substantial global challenge for the poultry industry concerning the surveillance of diverse viruses. In addition, gammaCoVs serve as a significant challenge when implementing strategic actions for the prevention and mitigation of poultry gammaCoVs outbreaks.

The genus *Deltacoronavirus* is classified into three subgenera—*Andecovirus*, *Buldecovirus* and *Herdecovirus*, which include seven ratified species, six of which represent groups of viruses that were found only in birds. Only one deltaCoV species Coronavirus HKU15, subgenus *Buldecovirus,* represents a group of viruses that were detected in birds and in mammalian species, including pigs, an Asian leopard cat (*Prionailurus bengalensis*) and a Chinese ferret badger (*Melogale moschata*). The *Coronavirus HKU15* isolated from pigs and other viruses of subgenus *Buldecovirus* have been shown to have similar genetic characteristics (PDCoV HKU15 is more than 96% similar to SpCoV HKU17 in 7 aminoacid sequences of ICTV domains, which are used for species demarcation) and structure, indicating possible transmission of the virus from birds to mammals^11–14^. It was subsequently shown by phylogenetic analysis that porcine deltacoronaviruses (deltaCoVs) are capable of interspecies transmission^13,15^. Furthermore, a recent study describes cases of human infections with porcine deltacoronavirus (PDCoV) after probable contact with domestic animals^16^.

In general, studies on gammaCoVs and deltaCoVs show that wild birds play an important role in their circulation. Coronaviruses have been detected in many orders of wild birds, such as ducks, geese and swans (*Anseriformes*), storks (*Ciconiiformes*), shorebirds and gulls (*Charadriiformes*), pigeons (*Columbiformes*), chickens, turkeys, quails (*Galliformes*), songbirds (*Passeriformes*), pelicans (*Pelecaniformes*) and parrots (*Psittaciformes*)^15,17^. In general, the percentage of virus carriers depends on the order and sometimes the type of bird. For example, in one of the studies conducted in Sweden, it was found that the ducks were the most prominent hosts, carrying 39% of the gammaCoVs and deltaCoVs isolated from free-ranging birds. One the other hand, the overall proportion of carriers was 18.7% among all the studied wild birds^17^. Studies conducted in Korea demonstrated a lower percentage of virus carriers (0.95%) among wild birds, but, as in the previous study in Sweden, wild ducks were the dominant carriers. Overall, most studies indicate that the highest percentage of positive samples was obtained from birds of the order *Anseriformes*^17,18^.

At this time however, there are still many important, open questions regarding the full range of avian host species, the seasonal and geographic distribution of viral circulation and the genetic diversity of coronaviruses in birds. Limited numbers of studies are available that reveal the as-yet uncharted complexities in the ecology and evolution of CoVs in birds^15^. In particular, in Russian Federation, there are only a few reported studies of poultry CoVs, and these studies investigated the IBV group.

Bochkov et al., 2006 presented a comprehensive genetic characterisation of AvCoVs isolated from 1998 to 2002 during a surveillance study that sampled more than 250 poultry farms^19^. The authors revealed the circulation of several different IBV genotypes originating from the AvCoV group, predominated by the Massachusetts genotype (including H120, H52 vaccine lines). The predominance of viruses from this genotype among poultry subsequently persisted until at least 2010 as was shown by another surveillance study of IBV, which is described in the publication by Ovchinnikova et al., 2011. The publication presents the results of molecular biological characterisation of AvCoVs isolated in Russia, as well as Ukraine and Kazakhstan in the period from 2007 to 2010^20^.

In effect, the surveillance and study of CoVs ecology and evolution contributes to the implementation of control measures in poultry agriculture^21^. Continued characterization of these viruses are also important for the prognosis of potential zoonotic spread of the viruses among mammalian species and humans. The aim of the work was to broaden the evaluation of ecology and diversity of CoVs in Russia. In this study we report the results of countrywide surveillance and the characterization of the genetic diversity of coronaviruses that were isolated from among wild birds and poultry in Russia in 2020.

## Materials and methods

### Ethics statements

The identification of bird species and their sampling was carried out by zoologists of regional laboratories of Federal Service for Surveillance on Consumer Rights Protection and Human Wellbeing (Rospotrebnadzor). Capture and sampling of birds were carried out in accordance with Russian legislation and under bioethics protocol № Vector/04-04.2018 issued by BioEthics Committee at FBRI SRC VB Vector Rosbotrebnadzor.

### Samples collection

Samples from wild birds and poultry were collected during the National Avian Viruses Surveillance Program, which supports sample collection from 48 regions of Russia. Samples were collected from the territories of 14 regions of the western part of Russia (Stavropol region, Astrakhan region, Rostov region, Republic of North Ossetia-Alania, Belgorod region, Kostroma region), Siberia (Novosibirsk region, Kurgan region, Tyumen region, Yamalo-Nenets Autonomous Okrug) and the Russian Far East (Kamchatka Krai, Primorsky Krai, Sakhalin region, Magadan region). Samples from birds included cloacal swabs, feces and internal organ fragments. The Copan Universal Transport Medium (UTM-RT) System (Copan Diagnostics Inc., USA) was used for sample collection. Samples (including cloacal swabs, droppings or internal organ fragments) were collected individually from 584 birds and used for the study. Swabs and organ homogenates were prepared as previously described^22^. Organ fragments were homogenized using Qiagen Tissue Liser LT (Qiagen, Germany). Swabs and organ homogenates were vortexed vigorously for 7–10 s and then centrifugated for 5 min at 12,000×g at 4 °C.

### One-step RT-PCR and nested PCR

Viral RNA was isolated from the collected samples using the RIBO-sorb or RIBO-prep kit (Interlabservice, Russia) according to the manufacturer’s instructions. With purpose of diagnostics of coronavirus, we used the published “modified PAN coronavirus PCR” and the test system developed in our laboratory using nested PCR^18,23^. All samples were analyzed using both methods. Modified PAN coronavirus PCR (one-step RT-PCR) was performed as described in Chamings et al. ^18^. Degenerate primers for the modified PAN coronavirus PCR (AC-CoV-F: GGTTGGGATTATCCWAARTGTG, AC-CoV-R: TGYTGTGARCAAAAYTCRTG) target the conserved region of the avian coronavirus polymerase gene (RdRp), producing a 602 bp amplicon. This test system allows for the detection of coronaviruses of all four genera (Alpha-, Beta-, Gamma- and Deltacoronaviruses). One step RT-PCR was performed using kits manufactured by Biolabmix LLC (Russia). These kits contain recombinant M-MuLV reverse transcriptase and DNA-dependent DNA polymerase in one enzyme mix. The reaction profile was as described in Chamings et al.^18^. Briefly, the reaction was performed at 50 °C for 20 min, 92 °C for 5 min, followed by 45 cycles of 93 °C for 30 s, with an annealing temperature for 30 s, 68 °C for 1 min, final 68 °C step for 3 min and held at 4 °C. The touchdown annealing temperature started at 60 °C for 3 cycles, then decreased by 2 °C every 3 cycles until 48 °C, which was used for the final 28 reaction cycles^18^. A test system was developed using nested PCR, which allows for detection with significantly increased sensitivity. DNA obtained from one-step RT-PCR using a modified PAN coronavirus PCR was used to perform nested PCR. The nested PCR test system included degenerate nested PCR primers for the detection of Gamma- and Deltacoronaviruses. The primers were made for the RdRp gene region, the coordinates of the nested primers were F: 14,203–14,224 and R: 14,607–14,629 and are given relative to the reference genome of the infectious bronchitis virus strain IBV D274 (GenBank ID: MH021175.1). The sequences of the primers were: Vec_CoVgd1 F2: CWAARTGTGAYAGRKCHATGCC, Vec_CoVgd1 R2: CCRTCRTCAGAMARDATCATNAR. The size of the amplicon with primers was 422 bp. For nested PCR, the BioMaster LR HS-PCR (2×) kit manufactured by Biolabmix (Russia) was used. This kit contains a mixture of HS-Taq DNA and Pfu DNA polymerases to ensure high fidelity amplification. The reaction profile was 94 °C for 3 min, 95 °C for 15 s, 50 °C for 30 s, 68 °C for 1 min for 40 cycles, then 68 °C for 7 min, held at 4 °C. The strain of chicken infectious bronchitis virus IBV D274 (GenBank ID: MH021175.1) was used as a positive control in all PCR performances. Purified water was used as a negative control in all PCR runs.

### Sequencing

The sequencing of coronavirus amplicons of the RdRp gene was performed using Illumina MiSeq. Library was made using a v3 reagent kit (Illumina, USA). Sequences were assembled by aligning reads with known references using bwa-0.7.15 software^24^. Obtained sequences were deposited in the GenBank database (NCBI) (Supplementary Table S1). Amplicon coverage is shown in Supplementary Table S1.

#### Phylogenetic analysis

Phylogenetic trees were constructed using partial sequences of RdRp gene obtained in this study and reference sequences of avian coronaviruses from NCBI GenBank representing all relevant clades. Multiple sequence alignment was performed using the MUSCLE alignment option of MEGA X software. Phylogenetic analysis was performed using the neighbour joining method with 1000 bootstrap replications using MEGA X software (http://www.megasoftware.net/).

## Results

### Detection of coronaviruses in poultry and wild birds in Russia

A total of 584 avian samples, including 393 from wild birds and 191 from domestic birds were collected and examined during surveillance within the territory of the Russian Federation in July–December 2020 (Table 1). Samples from wild birds were collected from 8 regions and samples from poultry were collected from 9 regions of Russia (Table 1). The sampled wild birds belonged to 31 species of 3 orders (Table 2). Most of the bird species belonged to the *Anseriformes* and *Charadriiformes* orders, which were represented in the study by 16 and 13 species, respectively. The order *Passeriformes* was represented by 2 species of wild birds. Wild bird samples were from Charadriiformes (n = 200, 13 species), Anseriformes (n = 180, 16 species) and Passeriformes (n = 13, 2 species). One-step RT-PCR analysis revealed that 70 of 584 samples (12,0%) were positive for coronavirus: 56 samples from wild birds (14.2% among wild birds) and 14 samples from poultry (7.3% among poultry). Coronaviruses were identified from 20 species of birds, which belonged to 4 orders. The wild bird, in 25 cases of coronavirus RNA detection belonged to the order *Anseriformes* (9 bird species), 28 cases—to the order *Charadriiformes* (8 bird species) and 3 cases—to the order *Passeriformes* (2 bird species). Coronavirus RNA was detected in fourteen chickens, order *Galliformes* (1 species) (Table 2).

**Table 1:** Results of the study of samples from wild and domestic birds.

No.	Region of samples collection	Number of samples		Number of positive samples							
				GammaCoV		DeltaCoV		Mixed gammaCoV + deltaCoV variants		Untyped	
		Wild*	Dom.*	Wild	Dom	Wild	Dom	Wild	Dom	Wild	Dom
1.	Novosibirsk region	26	–	3	–	1	–	–	–	6	–
2.	Yamalo-Nenets Autonomous Okrug	175	–	8	–	3	–	2	–	17	–
3.	Kamchatka Krai	125	–	–	–	3	–	4	–	7	–
4.	Primorsky Krai	6	–	1	–	–	–	–	–	–	–
5.	Sakhalin region	27	60	–	7	–	–	–	–	–	–
6.	Stavropol region	8	10	1	–	–	–	–	–	–	–
7.	Astrakhan region	–	20	–	1	–	–	–	–	–	–
8.	Magadan region	23	60	–	–	–	–	–	–	–	–
9.	Kurgan region	–	15	–	–	–	–	–	–	–	–
10.	Tyumen region	–	2	–	–	–	–	–	–	–	–
11.	Kostroma region	–	10	–	–	–	–	–	–	–	–
12.	Rostov region	–	4	–	–	–	–	–	–	–	–
13.	Republic of North Ossetia-Alania	3		–	–	–	–	–	–	–	–
14.	Belgorod region	–	10	–	2	–	–	–	–	–	4
Total		393	191	13	10	7	–	6	–	30	4

*Wild—samples from wild birds; dom.—samples from poultry.

**Table 2:** Overview of the bird species and prevalence of coronaviruses.

No.	Order		Species	No. of samples	No. of positive samples	Sequenced (MiSeq)	No. of viruses detected			Detected mixed variants in one sample*
			Wild birds				GammaCoV		DeltaCoV	
1.	Anseriformes		Common teal (*Anas crecca*)	41	2	1	1			
2.			Eurasian wigeon (*Mareca penelope*)	30	3	2	1		1	
3.			Mallard (*Anas platyrhynchos*)	28	3	2	3			2 GammaCoVs
4.			Gadwall (*Mareca strepera*)	16	4	2	1		3	GammaCoV + 2 DeltaCoVs
5.			Tufted duck (*Aythya fuligula*)	16	3	1	1			
6.			Northern pintail (*Anas acuta*)	12	4	1	1			
7.			Common merganser (*Mergus merganser*)	8	1	1	1			
8.			Greater scaup (*Aythya marila*)	7						
9.			Common scoter (*Melanitta nigra*)	7	4	1	2			2 GammaCoVs
10.			Greater white-fronted goose (*Anser albifrons*)	3						
11.			Mute swan (*Cygnus olor*)	3						
12.			Northern shoveler (*Spatula clypeata*)	3	1	1	1			
13.			Velvet scoter (*Melanitta fusca*)	2						
14.			Smew (*Mergellus albellus*)	2						
15.			Common pochard (*Aythya ferina*)	1						
16.			Bean goose (*Anser fabalis*)	1						
17.	Charadriiformes		Dunlin (*Calidris alpina*)	40	9	4	2		7	2 DeltaCoVs; GammaCoV + 2 DeltaCoVs; GammaCoV + 2 DeltaCoVs
18.			Siberian gull (*Larus fuscus heuglini*)	34	4	1	1		1	GammaCoV + DeltaCoV
19.			Slaty-backed gull (*Larus schistisagus*)	30	1	1			1	
20.			Black-headed gull (*Chroicocephalus ridibundus*)	28	2	1	1			
21.			Common gull (*Larus canus*)	21						
22.			Herring gull (*Larus argentatus*)	13	5	1	2			2 GammaCoVs
23.			Red-necked stint (*Calidris ruficollis*)	9	4	2	2		3	Gamma + 2 Delta; Gamma + Delta
24.			Common tern (*Sterna hirundo*)	8						
25.			Pomarine jaeger (*Stercorarius pomarinus*)	7	2	1			1	
26.			Common sandpiper (*Actitis hypoleucos*)	5	1	1	1			
27.			Eurasian woodcock (*Scolopax rusticola*)	3						
28.			Great knot (*Calidris tenuirostris*)	1						
29.			Broad-billed sandpiper (*Calidris falcinellus*)	1						
30.	Passeriformes		Hooded crow (*Corvus cornix*)	7	1	1			1	
31.			Rook (Corvus frugilegus)	6	2	1	2			2 GammaCoVs
**Poultry**										
32.	Galliformes		Chicken (*Gallus gallus domesticus*)	134	14	10	11			2 GammaCoVs
33.			Turkey (*Meleagris gallopavo domesticus*)	10						
34.	Anseriformes		Domestic duck (*Anas platyrhynchos domesticus*)	33						
35.			Domestic goose (*Anser anser domesticus*)	14						
	4 orders		36 species	584	70	36	34	18		

*“GammaCoV + 2 DeltaCoVs” means that one gammacoronavirus and two different deltacoronaviruses were detected in one avian sample; “2 Gamma” means that two different gammacoronaviruses were detected in one avian sample etc.

Of the total number of samples from individual birds positive for coronaviruses, molecular genetic analysis was carried out for 36 samples (26 samples from wild birds, 10—from poultry). These samples were from all studied geographic locations and avian species. We performed partial sequencing of a conservative region for all coronavirus genera, specifically that of the RdRp gene (around 400 bp) using NGS. The nucleotide sequences of 51 viruses were deposited in NCBI GenBank, and their accession numbers are listed in Supplementary Table S1. The sequence length of the RdRp gene of one virus was not sufficient to deposit in GenBank, and it was only used for genotyping.

The 52 coronaviruses, which were identified, included 34 gammaCoVs (23 in wild bird, 11 in poultry) and 18 deltaCoVs (Table 2, Supplementary Table S1). Thirteen deltaCoVs were detected among birds of the order *Charadriiformes* and 4 deltaCoVs were detected among the birds of the order *Anseriformes*. The use of NGS made it possible to identify the presence of multiple coronaviruses in one positive sample. The simultaneous presence of genetic material of different coronaviruses in a single sample was detected from 12 avian hosts. The simultaneous presence of both gamma- and deltacoronaviruses isolated from a single bird host was detected in 6 cases in wild birds, representing 23% of all wild bird cases. (Table 2) (23% of all sequenced wild birds).

Evolutionary relationships were determined by phylogenetic analysis of the obtained nucleotide sequences, together with reference sequences. This phylogenetic analysis included sequences of the same region of the RdRp gene of the ratified species and of other relevant reference coronaviruses from GenBank.

### Phylogenetic analysis of gammacoronaviruses in poultry and wild birds

**Figure 1:**
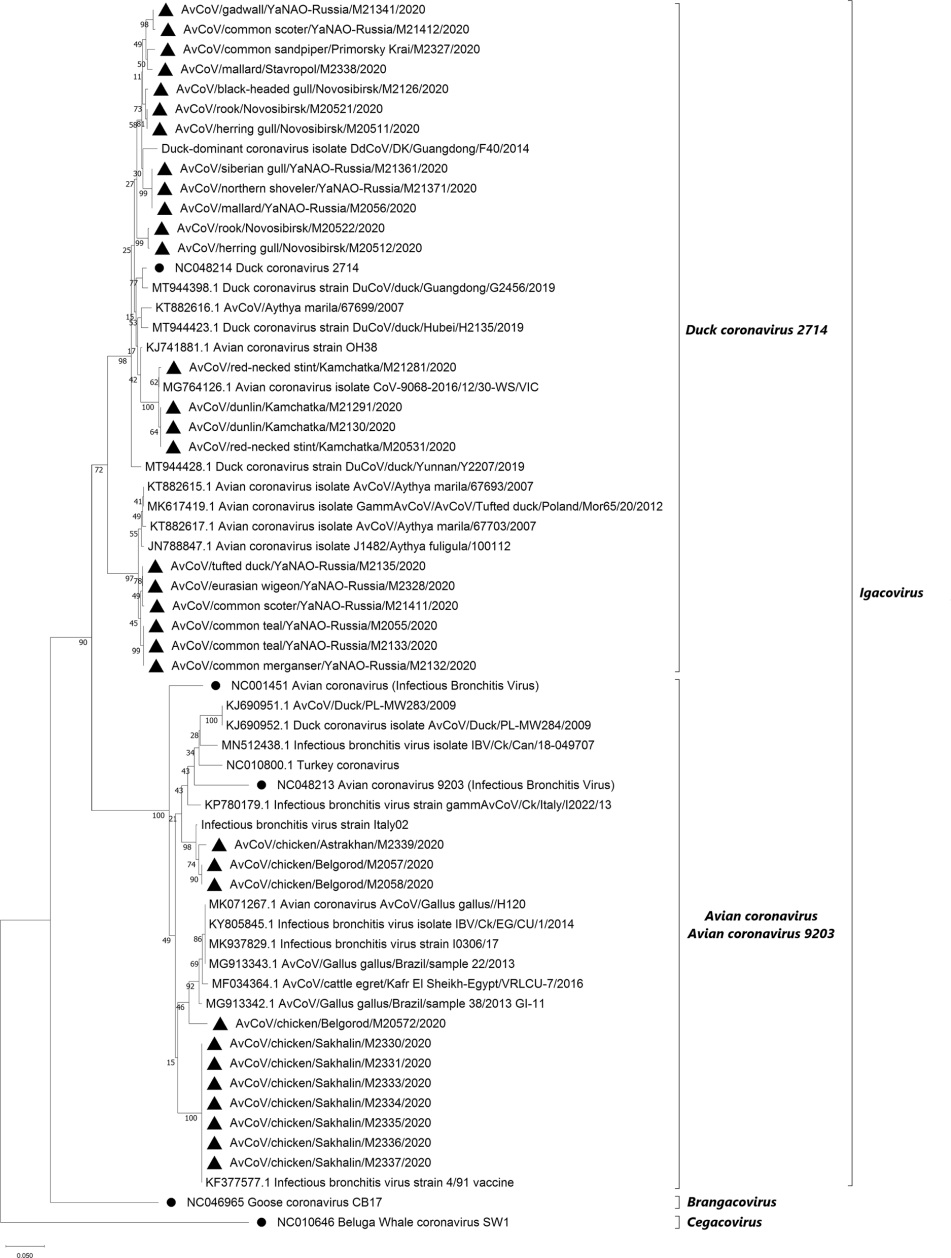
The phylogenetic analysis of the 360 base-pair region of the RdRp gene of select members of gammacoronaviruses by neighbour joining method using 1000 bootstraps. New viruses isolated within Russia are marked with black triangles. Ratified species (ICTV) are marked with black circles. The scale bar refers to a phylogenetic distance of 0.05 nucleotide substitutions per site.

The phylogenetic analysis of the gammaCoVs identified in this study showed that viruses belong to the subgenus *Igacovirus* and were categorized into two groups—the AvCov/AvCov9203 group and the *Duck coronavirus 2714* (DCoV 2714) group (Fig. 1).

All coronaviruses identified from poultry were grouped with the viruses of AvCov/AvCov9203 species. Partial sequences of the RdRp gene of gammaCoVs detected among chickens in the Astrakhan and Belgorod regions were classified into the group with infectious bronchitis virus strain Italy02 genotype and one virus grouped together with viruses of Massachusetts genotype (Mass genotype, strain AvCoV/Gallus gallus/H120). GammaCoVs detected in chickens in the Sakhalin region grouped with infectious bronchitis vaccine strain 4/91 (Fig. 1).

All gammaCoVs identified among wild birds in Russia belonged to the DCoV 2714 species group of subgenus *Igacovirus* (Fig. 1). The viruses belonging to the DCoV 2714 group identified in the study were further divided into two tentative subgroups. One subgroup included viruses that showed a high degree of identity with DCoV 2714 ratified species. The second tentative subgroup of viruses included CoVs detected in the Yamalo-Nenets Autonomous Okrug (YaNAO). This tentative subgroup includes only four viruses previously published in the literature and/or sequence repositories: AvCoV/Aythya marila/67693/2007, AvCoV/Aythya marila/67703/2007, GammaCoV/AvCoV/Tufted_duck/Poland/Mor65/20/2012, J1482/Aythya fuligula/100112 (Fig. 1). The nucleotide sequences of the RdRp gene fragment from the viruses within this subgroup were 91% or less similar to other viruses of the DCoV 2714 group (Supplementary Table S2).

### Phylogenetic analysis of deltacoronaviruses in wild birds

**Figure 2:**
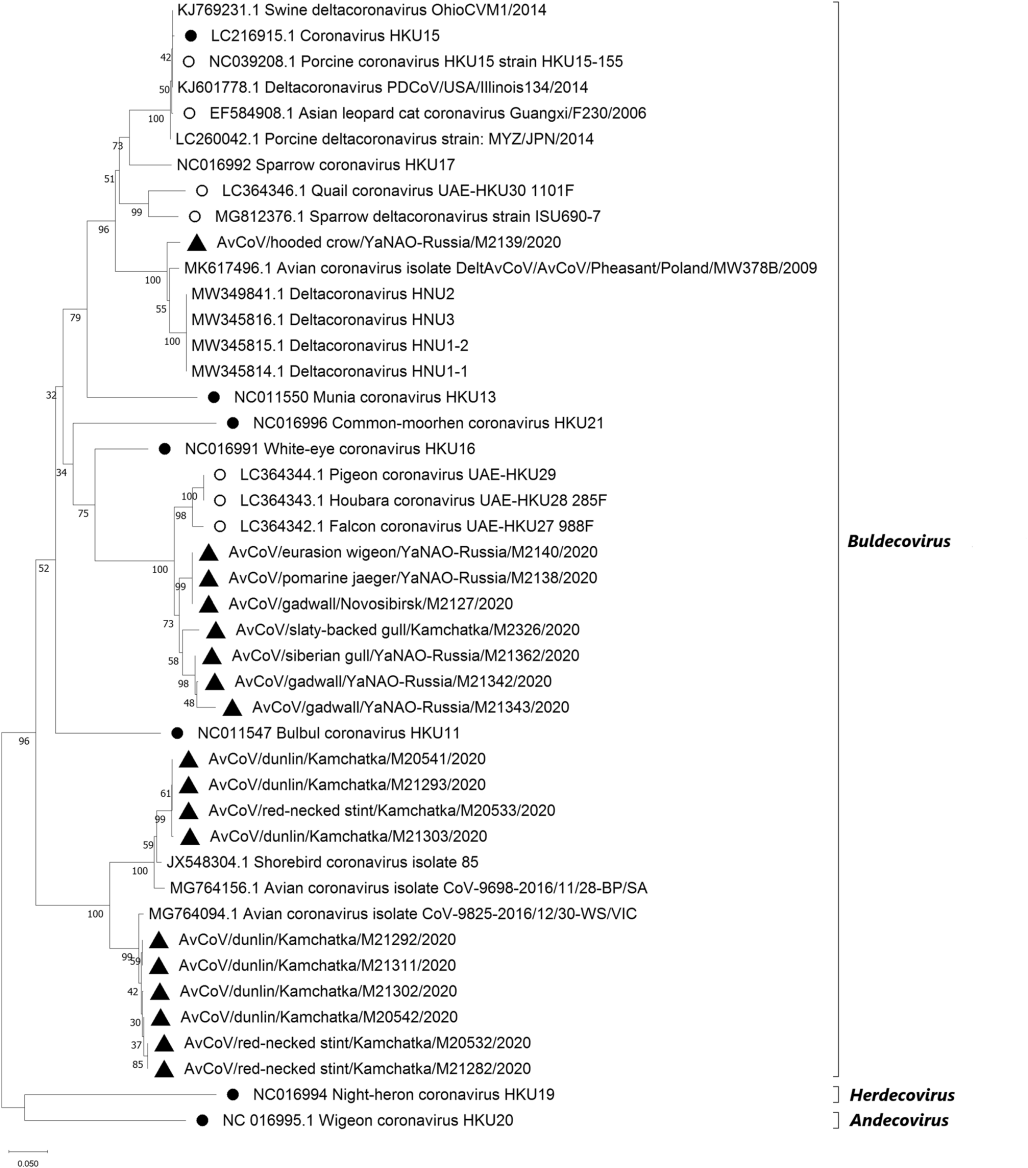
The phylogenetic analysis of a 357 base-pair region of the RdRp gene of select members of deltacoronaviruses by neighbour joining method using 1000 bootstraps. New viruses isolated in Russia are marked with black triangles. Ratified species (ICTV) are marked with black circles and other reference viruses with complete, published genome sequence is marked with empty circles. The scale bar refers to a phylogenetic distance of 0.05 nucleotide substitutions per site.

The phylogenetic analysis based on the partial sequences from the RdRp gene of deltaCoVs identified in this study showed that the viruses belonged to the subgenus *Buldecovirus* (Fig. 2). One of the studied viruses belonged to the group represented by the ratified species *Coronavirus HKU15* isolated from swine. Seven of the studied viruses tentatively grouped with the ratified species *White-eye coronavirus HKU16,* strain HKU16-6847 (Fig. 2). These 7 viruses were identified from the territory of the Kamchatka Krai, the Novosibirsk region, and the YaNAO, respectively, all belonged to the subgroup that includes previously identified three viruses. In addition, whole genome sequences have been obtained for these three viruses: Falcon coronavirus UAE-HKU27 988F (GenBank ID: LC364342.1), Pigeon coronavirus UAE-HKU29 (GenBank ID: LC364344.1), and Houbara coronavirus UAE-HKU28 285F (GenBank ID: LC364343.1). Ten viruses, whose circulation was detected from the territory of the Kamchatka Krai among birds of the order *Charadriiformes,* are grouped into a separate tentative subgroup of subgenus *Buldecovirus*, which includes a virus named Shorebird coronavirus isolate 85 (GenBank ID: JX548304.1). Nucleotide sequences of the RdRp gene fragment of these viruses share 80% or less sequence identity with the closest homologous ratified species and closest species, for which the complete genome is available (Supplementary Table S3).

## Discussion

### Avian coronavirus surveillance in Russia

The reported coronavirus surveillance study among wild and domestic birds was conducted using samples that were collected from several regions that cover a large territory of Russia—from the western regions to the territories of the Russian Far East. The aforementioned surveillance of CoVs was performed for the first time in wild birds in Russia. Analysis of the samples showed that coronaviruses are circulating among wild and domestic birds across various geographic regions. In total, the percentage of avian hosts that were shedding virus in our studies was 12% for the studied birds in Russia (70 out of 584 birds sampled). The percentage of detection of coronaviruses among wild birds was 14.2%, and for poultry 7.3%. Such a high percentage of avian viral hosts that were actively shedding virus identified in our study is consistent with previously reported surveillance data from other countries. A study conducted in Sweden was found that among wild ducks, the percentage of virus carriers reached 18.7%^17^ and in Australia it was 15.3% among wild birds^18^. The diversity of birds (including one domestic species), among which coronaviruses were identified, was represented by four orders and 20 species. The orders *Anseriformes* and *Charadriiformes* contributed the greatest proportion of viral RNA to this study. Only gammaCoVs were detected among poultry, while both gammaCoVs and deltaCoVs were detected among wild birds. The detection of deltaCoVs in wild birds in Russia is reported for the first time, to our knowledge.

Ten of 18 deltaCoVs were isolated from birds of the order *Charadriiformes*. Among the confirmed samples with co-infecting avian CoVs, 5 of the 12 samples also belonged to the order *Charadriiformes* (Table 2).

No circulation of alphacoronaviruses or betacoronaviruses was detected among our sampled birds, which, together with previously reported studies from other countries, which also did not detect alphacoronaviruses and betacoronaviruses in birds^4,15^, may be a preliminary indication that birds do not currently spread the coronaviruses or that these viruses have not been detected in birds up-to-date. However further monitoring is required due to the ability of these viruses to cross species barriers and due to potential danger of these viruses to human hosts^4,25^.

### Simultaneous presence of gammacoronaviruses and deltacoronaviruses from a single avian host

The coronavirus surveillance in wild and domestic birds within the territory of the Russian Federation in July–December 2020 revealed that a 12% of birds (70 of 584 sampled) carry a variety of deltaCoVs and gammaCoVs.

NGS analysis showed simultaneous presence of genetic material of different gammaCoVs and deltaCoVs from a single avian host in a 12 cases, six of which contained genetic material from viruses of both gammacoronavirus and deltacoronavirus genera. In most previous studies, the genetic characterization of CoVs from collected samples, i.e. swabs or organ homogenate, was performed using Sanger sequencing^15,18,26^. Unfortunately, Sanger sequencing does not allow for the characterization of two or more viruses in one from a single sample using PCR products. To overcome that limitation, in a study of coronaviruses in quail in Brazil, the cloning of PCR products from individual samples was used and obtained clones were sequenced. This protocol allowed for the detection of the viral RNA from *Gammacoronavirus* and *Deltacoronavirus* genera in one sample^27^. The limited number of publications detailing similar methods suggests that our study is among the first to demonstrate the simultaneous presence of different CoVs in the same bird. Our study demonstrated that the application of NGS is an effective and expedient way of conducting a more comprehensive characterization of surveillance samples, which may contain mixed populations of viruses and pseudo-viruses. The recently published and extensive genome analysis of *Coronaviridae* family representatives revealed the occurrence of recombination events. These recombination events often involve genomes of closely related viruses of the same subgenera, and in some rare cases, from different subgenera^28,29^. Although recombination events have not yet been detected among genomes of viruses belonging to different genera, genome analysis has suggested that such non-homologous recombination events may be possible. In addition, it has been demonstrated that genome recombination events may even occur among CoVs and highly dissimilar viruses such as toroviruses, gamma- and deltainfluenza viruses, reoviruses, rotaviruses, astroviruses and may even involve genetic material of infected hosts^28^. These findings highlight the need for more comprehensive surveillance of CoVs involving methods for evaluation of mixed populations of coronaviruses in various hosts.

### Gammacoronaviruses in poultry

All gammaCoVs identified in this study in poultry belonged to two groups represented by viruses from three IBV genotypes: Massachusetts and Italy02 in the western part of Russia, and the 793/B genotype in Sakhalin region. These three genotypes belong to AvCov/AvCov9203 group of subgenus *Igacovirus*. Coronaviruses of the Massachusetts lineage (first reported in USA) and the 793/B (Europe origin) genotypes are not only found worldwide, but they are the most well-characterised and important IBV genotypes due to their agricultural significance^21,30^. Genotype 793/B emerged in the UK and France in the 1990s, and became prevalent in Europe and many other parts of the world^31^. Coronaviruses of Italy 02 genotype were prevalent in Europe a few years ago (in 2002–2004) but are now in decline according to surveillance reports^31,32^. The infectious bronchitis caused by IBV is a serious poultry disease that may include symptoms such as poor weight gains, coughing, sneezing, egg-laying disorders^33^ and it is associated with serious economic consequences among unvaccinated flocks^21,34^. Vaccination is used as an effective measure for the protection of poultry from avian infectious bronchitis. The avian infectious bronchitis vaccine based upon the Massachusetts genotype viruses are produced and used globally, e.g. based on H120 strain. In addition, vaccines based upon viruses of the 793/B genotype, e.g. vaccine strain 4/91, are available and used in many countries^31,35^.

In previously reported studies (1998–2002, 2007–2010) several IBV genotypes, including the ones genetically characterised from this study, were registered in poultry in Russia. The prevalence and geographical distribution of the detected genotypes varied in different time periods^19,20^. Data from these and our studies suggests some differences in the spatial distribution of gammaCoVs that are currently circulating in poultry in Russia. The practical importance of research on CoVs for poultry-based agriculture is very high, and such monitoring data is valuable for both risk assessment and the implementation of protective measures. Such protective measures include vaccine development and implementation of region appropriate vaccination strategies.

### Gammacoronaviruses in wild birds

All gammaCoVs that were detected from wild birds in this study belonged to two subgroups in the DCoV 2714 group of viruses of subgenus *Igacovirus.* To our knowledge, this is the first report of detection of DCoV 2714 viruses in Russia. One subgroup of DCoV 2714 gammaCoVs was represented by ratified species *Duck coronavirus 2714*, which was previously identified in China in 2014^36^. The second subgroup differed significantly from other group of the DCoV 2714 viruses. In this subgroup, only four viruses have been identified and subsequently published: two in Sweden, one from Poland, and one from Hong Kong^17,26,37^. Similar to this study, a published analysis of strains of this subgroup from Sweden showed 92% or less identity of the ORF1ab gene fragment with strains that were deposited in Genbank in 2016^17^. These data tentatively indicate that these viruses may belong to a new subgroup of gammaCoVs, which are currently not represented by a ratified species.

According to previous studies, gammaCoVs may be transferred between wild birds and poultry. The occurrence of the virus transfer from poultry to wild birds was reported not only for wild type variants, but also for vaccine strains of IBV^38,39^. Overall, the circulation of a fairly wide variety of gammaCoVs both among wild and domestic birds was detected on the territory of the Russian Federation with potential importance for agriculture.

### Deltacoronaviruses in wild birds

The circulation of CoVs that shared sequence similarity with the genus *Deltacoronavirus* was only detected only among wild birds in this study. This is consistent with the literature, which shows that all previously investigated deltaCoVs have been found in wild birds^14,15^. The deltaCoVs detected in Russia and reported upon in this study belonged to the subgenus *Buldecovirus*. Phylogenetic analysis showed that the viruses belonged into three separate groups of *Buldecovirus* subgenus*.* One group of deltaCoVs represented in Russia is in the same branch as ratified species *White-eye coronavirus HKU16*. These group included previously characterized viruses, which had their complete genome sequenced (Falcon coronavirus UAE-HKU27, Houbara coronavirus UAE-HKU28, Pigeon coronavirusUAE-HKU29). The analysis of the viruses showed that they are less than 90% similar in amino acid sequence to *White-eye coronavirus HKU16* and other established viruses classified as deltaCoVs^12^. Ten viruses that were isolated from avian hosts in this study from the territory of the Kamchatka Krai are grouped into a separate branch, which differs from all established species (Supplementary Table S3). This group does not have previously characterized viruses from which whole genome sequences have been obtained. This group includes a previously reported virus named Shorebird coronavirus isolate 85 (GenBank ID: JX548304.1), isolated from the Ruddy turnstone (*Arenaria interpres*) in 2004, which was isolated in the United States. This virus has been described as a new deltaCoV^40^. The distinctiveness of the group is supported by phylogenetic analysis reported in previous studies^26^. However more genomic sequence for the viruses in this group is required for the designation of a new established species according to ICTV criteria. It should be noted that this group contains viruses that have been detected only among birds of order *Charadriiformes*, which includes the viruses identified during the course of this study in Russia, as well as viruses identified in other countries, including Poland, Australia and the USA^15,18,26^. One of the viruses identified in the current study belonged to the group that is represented by the ratified species *Coronavirus HKU15* isolated from swine (PDCoV)^13^*.* This is the only group in genus *Deltacoronavirus*, which, in addition to avian viruses, contains viruses detected and in some cases, isolated from mammalian species (e.g. pigs, asian leopard cat, ferret badger) from many countries, including China, South Korea, Japan, Poland and the USA^15,26^. Analysis of the genomes of the viruses in the PDCoV group suggested a recent event of zoonotic transmission from birds to mammals^13,15^. Global spread of the viruses of this group in birds and various mammalian species, including a recent report of human infection demonstrate that these viruses may be concern for human health^15,16^.

### Wild bird species involved in spreading coronaviruses

In this study, we examined 393 samples from wild birds belonging to 31 species. 380 samples were collected from birds of the *Anseriformes* and *Charadriiformes* orders and 13 samples were collected from birds of the *Passeriformes* order. The proportion of birds actively shedding viruses were the same (14%) for the birds of *Anseriformes* and *Charadriiformes* orders. GammaCoVs and deltaCoVs were detected from birds of both orders. These data suggest that birds of these orders play important role in the circulation of CoVs. These results are similar to previously reported studies that also showed important role of the birds of Anseriformes and Charadriiformes orders in avian CoVs ecology^41,42^.

In this study, the CoVs have been also detected among species such as Rook (*Corvus frugilegus*) and Hooded crow (*Corvus cornix*) from the order *Passeriformes*. It is worth noting that in this study, a virus from the species of *Coronavirus HKU15*, which have been isolated from both birds and mammals, was detected in a Hooded crow. In prior studies, PDCoVs were detected in sparrows (*Passer domesticus*), which belong to order *Passeriformes*^15^. The order *Passeriformes* is extremely important in the epidemiology and epizootiology of coronaviruses, since its representatives are often synanthropic species whose lifestyles are often associated with human activities and mayincludes close contacts with people and domestic animals^15^. The role of this taxonomic group in CoVs carriage and transmission need to be further evaluated.

Most bird species of the *Anseriformes* and *Charadriiformes* orders, among which we have reported upon the circulation of AvCoVs, undertake long-distance migration routes in Eurasia, twice per year^43,44^. For example, the Gadwall (*Mareca strepera*) migrates to the coastal regions of the Mediterranean and Black Seas, East Africa, the Caspian coast, Iran and India^45^. Eastern populations migrate to the east and southeast, reaching the territories of southeast Asia and the Japanese islands^46^. The nesting area of various populations of the Common teal (*Anas crecca*), a member of the *Anatidae* family, is extensive^47^. It inhabits much of western Europe and most of central Asia to northern Iran, northwestern Mongolia and Northeast China, and includes northern Japan and the western part of North America to the Great Lakes^47^. The spectacular migrations undertaken by these species suggest that the possibility exists for the intermingling of AvCoV viral RNA where these bird species are co-located^43–46^.

## Conclusion

A comprehensive surveillance of various avian coronaviruses among wild and domestic birds was conducted in several regions of the Russian Federation in July–December 2020. This was the first study that included the monitoring of the circulation of CoVs in wild birds in Russia. The study revealed the broad circulation of various coronaviruses in Russia, which are shed and potentially transmitted by birds. This is also the the first study to report a considerable number^6^ of cases that simultaneously detected the shedding gammaCoVs and deltaCoVs from a single avian host. Viruses detected among wild and domestic birds belonged to groups of established species of gammaCoVs, i.e. AvCov/AvCov9203 group in poultry, DCoV 2714 group in wild birds, and deltaCoVs, which arecurrently designated by *Coronavirus HKU15* and *White-eye coronavirus HKU16*. Genetic diversity of coronaviruses in wild birds was detected within genus *Gammacoronavirus* (DCoV 2714 group) and in genus *Deltacoronavirus* (HKU16 group). To our knowledge, this is the first report of the circulation of deltacoronaviruses among wild birds in Russia. No circulation of alphacoronaviruses or betacoronaviruses was detected among domestic or wild birds. Further monitoring is required due to the ability of these viruses to cross species barriers. Similarly to global trends, this study demonstrated the important role of wild birds of the *Anseriformes* and *Charadriiformes* orders in the circulation of gammaCoVs and deltaCoVs in Russia. Continuous surveillance of these viruses and further characterisation of avian coronaviruses will contribute to a better understanding of coronavirus spread and variability. This study underscores and provides critical data to the literature that could potentially be of great importance for epidemiological and epizootiological control within ecologically and agriculturally relevant species.

## Supplementary Information

Supplementary Information.

## Data Availability

All relevant data are within the paper and its Supporting Information files.
