# Supplementary material for: Diversity of gammacoronaviruses and deltacoronaviruses in wild birds and poultry in Russia
Source: Sci Rep. 2022 Nov 12;12:19412. doi: 10.1038/s41598-022-23925-z (PMC9653423; doi:10.1038/s41598-022-23925-z)
Supplement: Supplementary file 1 — Supplementary Information. [file 41598_2022_23925_MOESM1_ESM.pdf]

Diversity of gammacoronaviruses and deltacoronaviruses in wild birds and poultry in Russia

Vasily Marchenko[1,*], Alexey Danilenko[1], Nataliya Kolosova[1], Maria Bragina[1], Marina Molchanova[1], Yuliya Bulanovich[1], Vladimir Gorodov[2], Sergey Leonov[2], Andrey Gudymo[1], Galina Onkhonova[1], Svetlana Svyatchenko[1], Alexander Ryzhikov[1]

1 – State Research Center of Virology and Biotechnology Vector Rospotrebnadzor, Koltsovo, 630559, Russia,

2 – Siberian Federal Scientific Centre of Agro-BioTechnologies, RAS, Novosibirsk, Russia

***marchenko_vyu@vector.nsc.ru**

SUPPLEMMENTARY INFORMATION

Supplementary Table 1. Summary of coronaviruses specimen data

образец	avian ID	Accesion ID	size	coverage	Taxonomic name	Sample type	Sampling location	Date
AvCoV/black-headed gull/Novosibirsk/M2126/2020	BN1	OM925573	546	52907	black-headed gull/gammacoronavirus	Cloacal swab	Novosibirsk region	20.10.2020
AvCoV/common scoter/YaNAO-Russia/M21411/2020	CY1	OM970096	547	33924	common scoter /gammacoronavirus	Cloacal swab	YaNAO	13.10..2020
AvCoV/common scoter/YaNAO-Russia/M21412/2020	CY1	OM970097	390	5850	common scoter/gammacoronavirus	Cloacal swab	YaNAO	13.10.2020
AvCoV/tufted duck/YaNAO-Russia/M2135/2020	TY1	OM970098	547	43829	tufted duck /gammacoronavirus	Cloacal swab	YaNAO	13.10.2020
AvCoV/common teal/YaNAO-Russia/M2133/2020	CY2	OM970099	547	56949	common teal/gammacoronavirus	Cloacal swab	YaNAO	13.10.2020
AvCoV/common merganser/YaNAO-Russia/M2132/2020	CY3	OM970100	547	27489	common merganser /gammacoronavirus	Cloacal swab	YaNAO	13.10.2020
AvCoV/gadwall/YaNAO-Russia/M21341/2020	GY1	OM970101	546	38686	gadwall /gammacoronavirus	Cloacal swab	YaNAO	13.10.2020
AvCoV/siberian gull/YaNAO-Russia/M21361/2020	SY1	OM970102	547	33789	siberian gull/gammacoronavirus	Cloacal swab	YaNAO	13.10.2020
AvCoV/northern shoveler/YaNAO-Russia/M21371/2020	NY1	OM970103	547	50795	northern shoveler /gammacoronavirus	Cloacal swab	YaNAO	13.10.2020
AvCoV/red-necked stint/Kamchatka/M21281/2020	RK1	OM970104	547	58124	red-necked stint/gammacoronavirus	Feces	Kamchatka Krai	23.10.2020
AvCoV/dunlin/Kamchatka/M21291/2020	DK1	OM970105	382	19753	dunlin/gammacoronavirus	Feces	Kamchatka Krai	23.10.2020
AvCoV/common teal/YaNAO-Russia/M2055/2020	CY4	OM970106	547	292	common teal/gammacoronavirus	Cloacal swab	YaNAO	13.10.2020
AvCoV/mallard/YaNAO-Russia/M2056/2020	MY1	OM970107	547	17540	mallard/gammacoronavirus	Cloacal swab	YaNAO	13.10.2020
AvCoV/rook/Novosibirsk/M20521/2020	RN1	OM970108	547	35942	rook/gammacoronavirus	Cloacal swab	Novosibirsk region	20.10.2020
AvCoV/rook/Novosibirsk/M20522/2020	RN1	OM970109	547	11894	rook/gammacoronavirus	Cloacal swab	Novosibirsk region	20.10.2020
AvCoV/chicken/Belgorod/M2057/2020	CB1	OM970110	547	4612	chicken/gammacoronavirus	Trachea	Belgorod region	02.10.2020
AvCoV/chicken/Belgorod/M2058/2020	CB2	OM970111	547	6977	chicken/gammacoronavirus	Lung	Belgorod region	02.10.2020
AvCoV/eurasian wigeon/YaNAO-Russia/M2328/2020	EY1	OM970112	382	106655	eurasian wigeon /gammacoronavirus	Cloacal swab	YaNAO	09.06.2020
AvCoV/common sandpiper/Primorsky Krai/M2327/2020	CP1	OM970113	546	124456	common sandpiper/gammacoronavirus	Intestines	Primorsky Krai	11.11.2020
AvCoV/chicken/Sakhalin/M2330/2020	CS1	OM970114	394	103378	chicken/gammacoronavirus	Trachea	Sakhalin region	05.06.2020
AvCoV/chicken/Sakhalin/M2331/2020	CS2	OM970115	394	107169	chicken/gammacoronavirus	Trachea	Sakhalin region	05.06.2020
AvCoV/chicken/Sakhalin/M2333/2020	CS3	OM970116	394	133485	chicken/gammacoronavirus	Trachea	Sakhalin region	11.12.2020
AvCoV/chicken/Sakhalin/M2334/2020	CS4	OM970117	547	126662	chicken/gammacoronavirus	Trachea	Sakhalin region	11.12.2020
AvCoV/chicken/Sakhalin/M2335/2020	CS5	OM970118	547	121335	chicken/gammacoronavirus	Trachea	Sakhalin region	11.12.2020
AvCoV/chicken/Sakhalin/M2336/2020	CS6	OM970119	547	110239	chicken/gammacoronavirus	Trachea	Sakhalin region	11.12.2020
AvCoV/chicken/Sakhalin/M2337/2020	CS7	OM970120	547	54609	chicken/gammacoronavirus	Trachea	Sakhalin region	11.12.2020
AvCoV/mallard/Stavropol/M2338/2020	MS1	OM970121	546	128073	mallard/gammacoronavirus	Cloacal swab	Stavropol region	11.12.2020

AvCoV/chicken/Astrakhan/M2339/2020	CA1	OM970122	382	99218	chicken/gammacoronavirus	Intestines	Astrakhan region	21.12.2020
AvCoV/dunlin/Kamchatka/M2130/2020	DK2	ON004931	547	48071	dunlin/gammacoronavirus	Feces	Kamchatka Krai	23.10.2020
AvCoV/herring gull/Novosibirsk/M20511/2020	HN1	ON605847	395	30886	herring gull/gammacoronavirus	Cloacal swab	Novosibirsk region	20.10.2020
AvCoV/herring gull/Novosibirsk/M20512/2020	HN1	ON605848	395	10201	herring gull /gammacoronavirus	Cloacal swab	Novosibirsk region	20.10.2020
AvCoV/red-necked stint/Kamchatka/M20531/2020	RK2	ON605849	395	8833	red-necked stint/gammacoronavirus	Feces	Kamchatka Krai	23.10.2020
AvCoV/chicken/Belgorod/M20572/2020	CB1	ON605853	395	322	chicken/gammacoronavirus	Trachea	Belgorod region	02.10.2020
AvCoV/red-necked stint/Kamchatka/M20532/2020	RK2	ON004916	579	35632	red-necked stint/deltacoronavirus	Feces	Kamchatka Krai	23.10.2020
AvCoV/red-necked stint/Kamchatka/M20533/2020	RK2	ON004917	579	9324	red-necked stint/deltacoronavirus	Feces	Kamchatka Krai	23.10.2020
AvCoV/dunlin/Kamchatka/M20541/2020	DK3	ON004918	579	20023	dunlin/deltacoronavirus	Feces	Kamchatka Krai	23.10.2020
AvCoV/dunlin/Kamchatka/M20542/2020	DK3	ON004919	579	3789	dunlin/deltacoronavirus	Feces	Kamchatka Krai	23.10.2020
AvCoV/gadwall/Novosibirsk/M2127/2020	GN1	ON004920	379	20	gadwall/deltacoronavirus	Cloacal swab	Novosibirsk region	20.10.2020
AvCoV/gadwall/YaNAO-Russia/M21342/2020	GY1	ON004921	379	9895	gadwall/deltacoronavirus	Cloacal swab	YaNAO	13.10.2020
AvCoV/gadwall/YaNAO-Russia/M21343/2020	GY1	ON004922	379	2708	gadwall/deltacoronavirus	Cloacal swab	YaNAO	13.10.2020
AvCoV/siberian gull/YaNAO-Russia/M21362/2020	SY1	ON004923	379	2356	siberian gull/deltacoronavirus	Cloacal swab	YaNAO	13.10.2020
AvCoV/slaty-backed gull/Kamchatka/M2326/2020	SK1	ON004924	379	115188	slaty-backed gull/deltacoronavirus	Feces	Kamchatka Krai	09.06.2020
AvCoV/red-necked stint/Kamchatka/M21282/2020	RK1	ON004925	379	1007	red-necked stint/deltacoronavirus	Feces	Kamchatka Krai	23.10.2020
AvCoV/dunlin/Kamchatka/M21292/2020	DK1	ON004926	542	27840	dunlin/deltacoronavirus	Feces	Kamchatka Krai	23.10.2020
AvCoV/dunlin/Kamchatka/M21293/2020	DK1	ON004927	379	7098	dunlin/deltacoronavirus	Feces	Kamchatka Krai	23.10.2020
AvCoV/dunlin/Kamchatka/M21302/2020	DK2	ON004928	542	2379	dunlin/deltacoronavirus	Feces	Kamchatka Krai	23.10.2020
AvCoV/dunlin/Kamchatka/M21303/2020	DK2	ON004929	542	15496	dunlin/deltacoronavirus	Feces	Kamchatka Krai	23.10.2020
AvCoV/dunlin/Kamchatka/M21311/2020	DK4	ON004930	542	35497	dunlin/deltacoronavirus	Feces	Kamchatka Krai	23.10.2020
AvCoV/eurasion wigeon/YaNAO-Russia/M2140/2020	EY2	ON605854	379	49598	eurasion wigeon/deltacoronavirus	Cloacal swab	YaNAO	13.10.2020
AvCoV/hooded crow/YaNAO-Russia/M2139/2020	HY1	ON605855	379	36621	hooded crow/deltacoronavirus	Cloacal swab	YaNAO	13.10.2020
AvCoV/pomarine jaeger/YaNAO-Russia/M2138/2020	PY1	ON605856	379	40254	pomarine jaeger/deltacoronavirus	Cloacal swab	YaNAO	13.10.2020

Supplementary Table S2. Identity matrix of the partial nucleotide sequence of the RdRp gene (360 nucleotides) of a tentative new clade in the duck line of gammacoronaviruses, identified species of gammacoronaviruses, representative viruses isolated in Russia, and viruses from studies in other countries.

	**AvCoV/common scoter/YaNAO-Russia/M21411/2020**	**AvCoV/tufted duck/YaNAO-Russia/M2135/2020**	**AvCoV/eurasian wigeon/YaNAO-Russia/M2328/2020**	**AvCoV/common teal/YaNAO-Russia/M2055/2020**	**AvCoV/common merganser/YaNAO-Russia/M2132/2020**	**AvCoV/common teal/YaNAO-Russia/M2133/2020**	MK617419.1 Avian coronavirus isolate Gamm8.aCoV/AvCoV/Tufted_duck/Poland/Mor65/20/2012	JN788847.1 Avian coronavirus isolate J1482/Aythya [illegible]	KT882615.1 Avian coronavirus isolate AvCoV/Aythya marila/67693/2007	KT882617.1 Avian coronavirus isolate AvCoV/Aythya marila/67703/2007	**AvCoV/red-necked stint/Kamchatka/M21281/2020**	MG764126.1 Avian coronavirus isolate CoV-9068-2016/12/30-WS/VIC	NC_048214.1 Duck coronavirus isolate DK/GD/27/2014	KT882616.1 AvCoV/Aythya marila/67699/2007	KJ741881.1 Avian coronavirus strain OH38	AvCoV/gadwall/YaNAO-Russia/M21341/2020	MK204411.1 Avian coronavirus	MH453802.1 AvCoV/Red-necked Avocet/MW10/2013	**AvCoV/siberian gull/YaNAO-Russia/M21361/2020**	**AvCoV/rook/Novosibirsk/M20522/2020**	DdCoV/DK/Guangdong/F40/2014	MK204393.1 Avian coronavirus	NC_001451.1 Avian infectious bronchitis virus
	1	2	3	4	5	6	7	8	9	10	11	12	13	14	15	16	17	18	19	20	21	22	23
1	ID	100	100	99	99	99	99	99	99	99	90	90	90	91	91	91	91	91	89	89	89	89	81
2	100	ID	100	99	99	99	99	99	99	99	90	90	90	91	91	91	91	91	90	90	89	89	81
3	100	100	ID	99	99	99	99	99	99	99	90	90	90	91	91	91	91	91	90	90	89	89	81
4	99	99	99	ID	100	100	99	98	99	98	90	90	90	91	91	91	91	91	90	89	90	89	81
5	99	99	99	100	ID	100	99	98	99	98	90	90	90	91	91	91	91	91	90	89	90	89	81
6	99	99	99	100	100	ID	99	98	99	98	90	90	90	91	91	91	91	91	90	89	90	89	81
7	99	99	99	99	99	99	ID	99	100	99	90	90	90	91	91	91	91	91	89	90	89	89	81
8	99	99	99	98	98	98	99	ID	99	99	90	90	90	91	91	91	91	91	89	90	89	89	80
9	99	99	99	99	99	99	100	99	ID	99	90	90	90	91	91	91	91	91	89	90	89	89	81
10	99	99	99	98	98	98	99	99	99	ID	90	90	90	91	91	90	91	91	89	89	89	89	81
11	90	90	90	90	90	90	90	90	90	90	ID	100	95	95	97	94	97	96	95	96	94	95	83
12	90	90	90	90	90	90	90	90	90	90	100	ID	96	96	98	94	97	96	95	96	94	95	83
13	90	90	90	90	90	90	90	90	90	90	95	96	ID	97	98	96	98	97	96	98	97	96	82
14	91	91	91	91	91	91	91	91	91	91	95	96	97	ID	98	95	98	97	96	96	94	96	81
15	91	91	91	91	91	91	91	91	91	91	97	98	98	98	ID	97	99	98	97	98	96	98	82
16	91	91	91	91	91	91	91	91	91	90	94	94	96	95	97	ID	96	95	98	96	96	95	81
17	91	91	91	91	91	91	91	91	91	91	97	97	98	98	99	96	ID	99	96	98	96	97	82
18	91	91	91	91	91	91	91	91	91	91	96	96	97	97	98	95	99	ID	95	96	95	96	81
19	89	90	90	90	90	90	89	89	89	89	95	95	96	96	97	98	96	95	ID	97	97	96	81
20	89	90	90	89	89	89	90	90	90	89	96	96	98	96	98	96	98	96	97	ID	96	96	82
21	89	89	89	90	90	90	89	89	89	89	94	94	97	94	96	96	96	95	97	96	ID	95	81
22	89	89	89	89	89	89	89	89	89	89	95	95	96	96	98	95	97	96	96	96	95	ID	81
23	81	81	81	81	81	81	81	80	81	81	83	83	82	81	82	81	82	81	81	82	81	81	ID

Supplementary Table S3. Identity matrix of the partial nucleotide sequence of the RdRp gene (357 nucleotides) of the tentative new deltacoronavirus clade and established deltacoronavirus species.

	**AvCoV/red-necked stint/Kamchatka/M20532/2020**	**AvCoV/red-necked stint/Kamchatka/M21282/2020**	**AvCoV/dunlin/Kamchatka/M20542/2020**	**AvCoV/dunlin/Kamchatka/M21302/2020**	**AvCoV/dunlin/Kamchatka/M21292/2020**	**AvCoV/dunlin/Kamchatka/M21311/2020**	MG764094.1 Avian coronavirus isolate CoV-	**AvCoV/red-necked stint/Kamchatka/M20533/2020**	**AvCoV/dunlin/Kamchatka/M21303/2020**	**AvCoV/dunlin/Kamchatka/M20541/2020**	**AvCoV/dunlin/Kamchatka/M21293/2020**	JX548304.1 Shorebird coronavirus isolate 85	White-eye coronavirus HKU16 strain HKU16-6847	LC364343.1 Houbara coronavirus UAE-HKU28 285F	Coronavirus HKU15	Bulbul coronavirus HKU11-934	Sparrow coronavirus HKU17 strain HKU17-6124	Common-moorhen coronavirus HKU21 strain	Munia coronavirus HKU13-3514	Night-heron coronavirus HKU19
	1	2	3	4	5	6	7	8	9	10	11	12	13	14	15	16	17	18	19	20
1	ID	100	99	99	99	99	99	89	89	89	89	92	80	76	76	76	74	74	74	69
2	100	ID	99	99	99	99	99	89	89	89	89	92	80	76	76	76	74	74	74	69
3	99	99	ID	100	99	99	99	89	89	89	89	91	80	76	76	76	75	75	73	69
4	99	99	100	ID	100	100	99	88	88	88	88	91	79	76	76	76	75	75	74	69
5	99	99	99	100	ID	100	99	89	89	89	89	91	79	76	76	76	75	74	74	69
6	99	99	99	100	100	ID	99	89	89	89	89	91	79	76	76	76	75	74	74	69
7	99	99	99	99	99	99	ID	88	88	88	88	90	80	76	76	76	75	74	73	69
8	89	89	89	88	89	89	88	ID	100	100	100	97	77	75	75	73	74	72	74	68
9	89	89	89	88	89	89	88	100	ID	100	100	97	78	75	74	73	74	72	73	68
10	89	89	89	88	89	89	88	100	100	ID	100	97	77	75	75	73	74	72	74	68
11	89	89	89	88	89	89	88	100	100	100	ID	97	77	75	75	73	74	72	74	68
12	92	92	91	91	91	91	90	97	97	97	97	ID	80	75	74	73	74	74	75	69
13	80	80	80	79	79	79	80	77	78	77	77	80	ID	82	79	78	80	78	77	68
14	76	76	76	76	76	76	76	75	75	75	75	75	82	ID	76	76	75	75	77	65
15	76	76	76	76	76	76	76	75	74	75	75	74	79	76	ID	78	90	73	80	69
16	76	76	76	76	76	76	76	73	73	73	73	73	78	76	78	ID	76	74	74	71
17	74	74	75	75	75	75	75	74	74	74	74	74	80	75	90	76	ID	76	79	68
18	74	74	75	75	74	74	74	72	72	72	72	74	78	75	73	74	76	ID	74	68
19	74	74	73	74	74	74	73	74	73	74	74	75	77	77	80	74	79	74	ID	67
20	69	69	69	69	69	69	69	68	68	68	68	69	68	65	69	71	68	68	67	ID
